# Stable isotope labelling and gene expression analysis reveal dynamic nitrogen-supply mechanisms for rapid growth of Moso bamboo

**DOI:** 10.1093/hr/uhaf062

**Published:** 2025-02-25

**Authors:** Junbo Zhang, Man Shi, Chenglei Zhu, Kebin Yang, Quan Li, Xiaoming Song, Zhimin Gao, Tingting Cao, Dezheng Zhu, Xinzhang Song

**Affiliations:** State Key Laboratory of Subtropical Silviculture, Zhejiang A&F University, Wusu Street No. 666, Lin'an District, Hangzhou 311300, China; Institute of Environment, Resource, Soil and Fertilizer, Zhejiang Academy of Agricultural Sciences, Desheng Middle Road No. 298, Jianggan District, Hangzhou 310021, China; State Key Laboratory of Subtropical Silviculture, Zhejiang A&F University, Wusu Street No. 666, Lin'an District, Hangzhou 311300, China; State Key Laboratory of Subtropical Silviculture, Zhejiang A&F University, Wusu Street No. 666, Lin'an District, Hangzhou 311300, China; College of Landscape Architecture and Forestry, Qingdao Agricultural University, Changcheng Road No. 700, Chengyang District, Qingdao 266109, China; State Key Laboratory of Subtropical Silviculture, Zhejiang A&F University, Wusu Street No. 666, Lin'an District, Hangzhou 311300, China; School of Life Sciences/Library, North China University of Science and Technology, Bohai Avenue No. 21, Caofeidian District, Tangshan, Hebei 063210, China; International Center for Bamboo and Rattan, Institute of Gene Science and Industrialization for Bamboo and Rattan Resources, Futong East Street No. 8, Chaoyang District, Beijing 100102, China; State Key Laboratory of Subtropical Silviculture, Zhejiang A&F University, Wusu Street No. 666, Lin'an District, Hangzhou 311300, China; State Key Laboratory of Subtropical Silviculture, Zhejiang A&F University, Wusu Street No. 666, Lin'an District, Hangzhou 311300, China; State Key Laboratory of Subtropical Silviculture, Zhejiang A&F University, Wusu Street No. 666, Lin'an District, Hangzhou 311300, China

## Abstract

Rapid growth of Moso bamboo (*Phyllostachys edulis*) shoots (offspring ramet) is primarily fuelled by nitrogen (N) derived from parent ramet and absorbed by rhizome roots. However, the extent to which each N source supports the growth of offspring ramet and the underlying molecular mechanisms of N transport remain unclear. Here, clonal fragments consisting of a parent ramet, an offspring ramet, and an interconnected rhizome were established in a Moso bamboo forest. Additionally, ^15^N isotope tracing and transcriptome profiling were conducted concurrently to quantify the N contribution from the parent ramet and rhizome roots to the offspring ramet, and to reveal the molecular mechanisms underlying N transport during rapid growth (i.e. early, peak, branching, and leafing stages). The N acquisition strategy of offspring ramet shifted from being primarily provided by the parent ramet (72.53%) during early stage to being predominantly absorbed by rhizome roots (69.85%) during the leafing stage. Approximately equal N contributions (45.82%–54.18%) from the parent ramet and rhizome roots were observed during peak and branching stages. *PeAAP29123* was identified as a key gene for N transport, being most closely correlated with ^15^N content. Biomolecular assays demonstrated that PeHDZ23987 could activate the expression of *PeAAP29123* via two types of HD-motifs. Overexpression of *PeHDZ23987* and *PeAAP29123* significantly enhanced N starvation tolerance in transgenic rice with significantly improved N uptake efficiency. Our findings clarify the pattern and mechanisms of N supply for the rapid growth of Moso bamboo offspring ramet and provide transcriptomic evidence for long-distance N transport between clonal ramets.

## Introduction

Bamboo belongs to the grass family, Poaceae, and is a popular ornamental plant with a long history of cultivation in many countries, where it holds high socioeconomic and cultural value [1]. Moso bamboo (*Phyllostachys edulis*) is a large, woody, clonal plant widely distributed in East and Southeast Asia. As a fast-growing species, Moso bamboo has gained increasing importance due to its high capacity for carbon sequestration [2, 3]. Currently, the species occupies a total area of 5.27 Mha in China, accounting for 84.02% of its global distribution [4, 5]. This bamboo can grow 10–20 m within 35–40 days after sprouting of the shoot (offspring ramet) [6]. Numerous studies have attempted to elucidate the mechanisms responsible for the rapid growth of Moso bamboo offspring ramets by exploring internode elongation from perspectives of tissue structure [7, 8], endogenous hormones [8, ,9], and molecular processes [10, 11]. However, little attention has been paid to the nutrient supply, which is crucial for maintaining a high growth rate. This oversight may be attributed to the high mortality rate and fragility of Moso bamboo offspring ramets in the field, and the complexity of the underground rhizome network in Moso bamboo forests, which pose significant challenges to *in situ* observation of nutrient supply.

Clonal integration is an intrinsic mechanism for regulating nutrient transport and allocation among Moso bamboo ramets, which plays a crucial role in the growth and development of offspring ramets, as it enables interconnected ramets to share resources including water, carbohydrates, and mineral nutrients via rhizomes [12, 13]. Potential limitations to the growth of offspring ramets, such as slow nutrient absorption and limited resource availability, can be mitigated through clonal integration. Previous studies proposed that parent ramet provides substantial carbohydrates and nutrients to connected offspring ramets via underground rhizomes during rapid growth [6, 14]. Additionally, isotope tracing results demonstrated an unequal nitrogen (N) translocation pattern between mature (3-year-old) and young (1-year-old) ramets of Moso bamboo, with more ^15^N being allocated to young ramets in winter [13]. These results suggest that parent ramet plays a critical role in the nutrient supply during the rapid growth of Moso bamboo offspring ramets. In this context, rhizomes act as a bridge for nutrient transport between parent and offspring ramets of Moso bamboo. However, rhizome roots mainly function in resource acquisition [6, 15, 16], and could serve as an additional nutrient donor for offspring ramets. Thus, parent ramet and rhizome roots can both provide offspring ramets with the mineral nutrition simultaneously. However, accurate *in situ* quantification of the contributions from the parent ramet and rhizome roots to the rapid growth of offspring ramets remains a significant challenge.

The processes of N metabolism and transport are crucial for plant development. Many studies have examined N-associated metabolism in Moso bamboo [17]. Nitrate serves as a key molecular signal for regulating N absorption and assimilation, with Class I and II *PeNLP*s playing significant roles in nitrate signalling [18]. Previous studies showed that *PeNRT*s and *PeAMT*s are crucial for N uptake and distribution in plants and may aid in root-to-stem N transport [19]. Furthermore, amino acid permease (AAP) genes are reportedly responsible for amino acid absorption and long-distance transport [20, 21]. In particular, the specialized roles of AAPs have been investigated in Arabidopsis and other species. For example, AtAAP2 is localized to the phloem and transfers amino acids in the xylem–phloem vascular bundles [22]. Similarly, *AtAAP8* is highly expressed in the leaf phloem and participates in the process of N transport from leaves to sink organs [23]. Consistently, *PsAAP1,* activated by the *AtAAP1* promoter of Arabidopsis, can increase N use efficiency (NUE) and total biomass in transgenic peas (*Pisum sativum*) [24]. Moreover, GmAAP6a has been reported to play an important role in soybean (*Glycine max*) under low N stress [25]. Numerous studies have demonstrated that N was also important during bamboo growth and development [18, 19]. A recent bioinformatics analysis identified 16 *PeAAP*s in Moso bamboo [26]; however, the detailed biological functions of these AAPs remain elusive. Despite recent progress in elucidating the genes and regulatory networks involved in N metabolism and transport in Moso bamboo, previous studies predominantly focused on single organs or individual bamboo ramets [18, 19]. These studies often overlooked the unique characteristics of Moso bamboo as a clonal plant, resulting in limited insights into the complex processes of N metabolism and transport among interconnected ramets.

To address this research gap, we established clonal fragments consisting of a parent ramet, an offspring ramet, and an interconnected rhizome in a Moso bamboo forest. The rapid growth period of Moso bamboo offspring ramet was divided into four stages (early, peak, branching, and leafing stages). *In situ*  ^15^N isotope tracing and transcriptomic analysis were used to quantify the contributions of the parent ramet and rhizome roots to the rapid growth of offspring ramet during the aforementioned stages and to explore the possible molecular mechanism of N transport between the parent and offspring ramets. This study aims to clarify the pattern and underlying molecular mechanisms of N supply during the rapid growth of Moso bamboo offspring ramet, thus providing novel insights to improve the productivity of Moso bamboo forests from a nutrient supply perspective.

## Results

### N transport and allocation in the clonal fragments

**Figure 1:**
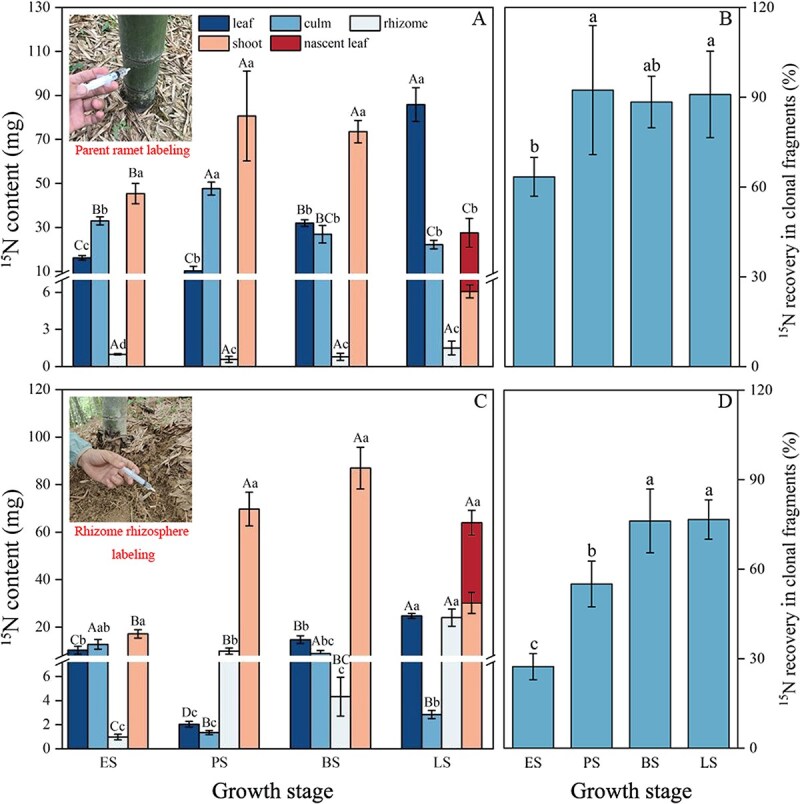
^15^N contents in different organs and ^15^N recovery within clonal fragment. (A, C) Changes in ^15^N contents in different organs during four growth stages when parent ramet and rhizome rhizosphere were labelled. Leaf and culm belong to parent ramet; shoot (culm of offspring ramet) and nascent leaf (only appeared during the leafing stage) belong to offspring ramet. Different uppercase and lowercase letters indicate significant differences among stages in each organ and significant differences among organs at each growth stage. (B, D) ^15^N recovery in clonal fragment during four growth stages when parent ramet and rhizome rhizosphere were labelled. Different lowercase letters indicate significant differences among different growth stages. ES, PS, BS, and LS represent the early, peak, branching, and leafing stage of rapid growth of offspring ramet, respectively. Data are shown as mean ± standard deviation (*n* = 3).

In ^15^N labelled parent ramets, ^15^N and total N (TN) contents in the culms decreased significantly. In contrast, they both gradually increased in the whole offspring ramet before the branching stage. Afterwards, leaf ^15^N and TN contents in the parent ramet increased, whereas ^15^N content in the whole offspring ramet decreased by nearly 50%, while TN content remained unchanged. Meanwhile, extremely low ^15^N and TN contents were observed in the rhizome, with no significant differences among growth stages (Fig. 1A, S1). After the early stage, ^15^N recovery in the clonal fragment reached as high as 90%, which was 30% higher than that detected in the early stage (Fig. 1B). When the rhizome rhizosphere was labelled with ^15^N, ^15^N content in the rhizome and whole offspring ramet significantly increased after the early stage (Fig. 1C). Interestingly, leaf and culm ^15^N contents in parent ramets significantly decreased during the peak stage but recovered during the branching stage. When the rhizome rhizosphere was labelled, the ^15^N recovery in the clonal fragment ranged from 27% during the early stage to 77% during the leafing stage, which was lower at each stage compared to when parent ramet was labelled (Fig. 1D).

### ^15^N fate in clonal fragment during rapid growth of Moso bamboo

**Figure 2:**
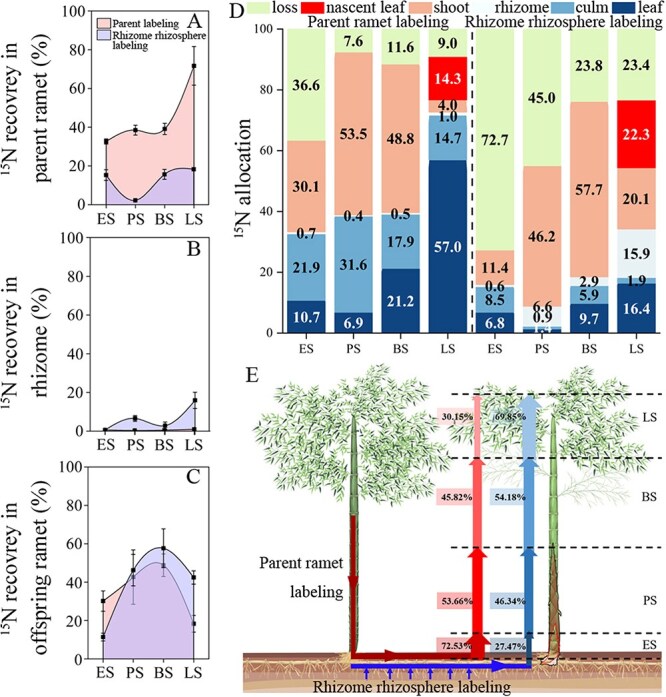
^15^N fate in clonal fragment when parent ramet and rhizome rhizosphere were labelled. (A–C) ^15^N recovery in parent ramet, rhizome, and offspring ramet. Data are shown as mean ± standard deviation (*n* = 3). (D) ^15^N allocation in different organs within clonal fragment. Leaf and culm belong to parent ramet, shoot (culm of offspring ramet) and nascent leaf (only appeared during the leafing stage) belong to offspring ramet, and loss represents unrecovered ^15^N. (E) The contributions of parent ramet and rhizome roots to the growth of offspring ramet. The percentages represent the supply ratio of N derived from the parent ramet and rhizome rhizosphere to the N content of the offspring ramet during each growth stage. The arrows indicate the source of ^15^N supply. ES, PS, BS, and LS represent the early, peak, branching, and leafing stage of rapid growth of offspring ramet, respectively.

When parent ramet was labelled, ^15^N recovery in it was significantly higher than that recorded when the rhizome rhizosphere was labelled during different growth stages (*P* < 0.05). Meanwhile, ^15^N recovery in the parent ramet increased rapidly after the branching stage, showing a 200% increase during the leafing stage compared to other growth stages (Fig. 2A). The ^15^N recovery in the rhizome was highest when the rhizome rhizosphere was labelled, ranging from 2.9% to 15.9% among the last three growth stages, which was higher than that recorded in the rhizome when the parent ramet was labelled (Fig. 2B). In addition, during the early stage, ^15^N recovery in the offspring ramet was 2.6 times higher when the parent ramet was labelled compared to when the rhizome rhizosphere was labelled (Fig. 2C). Further, during the peak and branching stages, ^15^N recovery in the offspring ramets was similar in the two ^15^N-labelling treatments. However, during the leafing stage, ^15^N recovery in the offspring ramet was highest when the rhizome rhizosphere was labelled, in which case it was 2.3 times higher than that recorded when the parent ramet was labelled. Interestingly, during the leafing stage, 30.4% of the ^15^N allocated to the offspring ramet before leafing stage was withdrawn from the leaves of the parent ramet when the parent ramet was labelled. Similarly, 15.3% of the ^15^N allocated to the offspring ramet before leafing stage was transferred to the rhizome when the rhizome rhizosphere was labelled (Fig. 2D). In addition, the supply ratio of N derived from the parent ramet and rhizome rhizosphere to the N content of the offspring ramet was ~7:3 at early stage, 5:5 at peak stage, 5:5 at branching stage, and 3:7 at leafing stage, respectively (Fig. 2E).

### Transcriptome sequencing and screening of target genes

**Figure 3:**
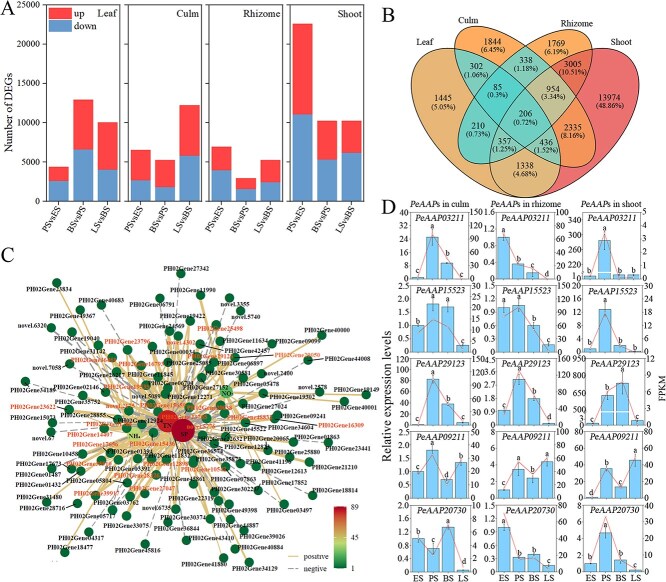
Screening of target genes for N transportation. (A) Number of DEGs in four organs among four growth stages. ‘Up’ and ‘down’ represent up- and downregulated genes. (B) Venn diagram of DEGs in the leaf, culm, rhizome, and shoot, between the peak and early stages of rapid growth. (C) Correlation network between common DEGs and physiological traits in leaves, culms, rhizomes, and shoots of Moso bamboo. The gene name in orange indicates that the gene is significantly correlated with four physiological traits. (D) RNA-Seq (broken line) and RT-qPCR (bar chart) data for the expression of nine genes in three different organs. Data are shown as mean ± standard deviation (*n* = 3). Different lowercase letters indicate significant differences among growth stages in each organ. ES, PS, BS, and LS represent the early, peak, branching, and leafing stage of rapid growth of offspring ramet, respectively. Leaf and culm belong to parent ramet, shoot (culm of offspring ramet) belong to offspring ramet.

To explore the molecular basis of N transport between clonal ramets of Moso bamboo, 48 samples of different organs from the four growth stages in the control treatment were used for transcriptome sequencing. Overall, 58 318 genes were identified, expressed during at least one developmental stage. A total of 50 936 genes were previously annotated in the Moso bamboo genome and were associated with various metabolic and cellular processes (Table S1). Principal component analysis (PCA) revealed a distinct separation of all leaf samples by the first principal component (PC1), while the second principal component (PC2) effectively distinguished offspring ramets during the early stage of growth (Fig. S2). During the rapid growth stage, 4376, 6500, 6924, and 22 605 differentially expressed genes (DEGs) were identified in leaves, culms, rhizomes, and shoots (culms of offspring ramet), respectively (Fig. 3A). ^15^N isotope results showed significant N translocation from the parent ramet to offspring ramet under both labelling treatments during the peak stage as compared to the early stage (Fig. 1). Meanwhile, the height of offspring ramet rapidly increased across these two stages (Fig. S3b). Therefore, DEGs between early and peak stages were further analysed. Specifically, 1445, 1844, 1769, and 13 974 genes were differentially expressed in leaves, culms, rhizomes, and shoots, respectively (Fig. 3B).

Two hundred six genes showed differential expression across four organs (Fig. 3B). Pearson correlation analysis between these 206 genes and physiological traits (TN, NH_4_^+^, NO_3_^−^, and soluble protein concentrations; Table S2) from various organs was performed. The results revealed that 26 genes were significantly correlated to four physiological traits (*P* < 0.05, Table S3). Further analysis revealed that *PeAAP29123* and *PeProT23796* were closely associated with these physiological traits (Fig. 3C; Table S3), and their homologues have been implicated in N transport. We speculate that the two genes may be involved in N uptake and transport. Additionally, nine DEGs were selected for quantitative real-time polymerase chain reaction (RT-qPCR) analysis, and the results showed high correlation between RNA sequencing (RNA-Seq) and RT-qPCR data indicating high reliability of the transcriptome data (Fig. 3D and Fig. S4). Additionally, function annotation results showed that a total of 1552 DEGs with ‘transport’ function were screened through the retrieval of gene annotations and Gene Ontology (GO) functions (Table S4), while 932 DEGs were enriched in the ‘transporter activity’ function (Fig. S5). Pearson correlation analysis between genes (FPKM ≥10) and the physiological data of various organs were conducted, and 41, 37, 31, and 59 DEGs were significantly correlated with physiological traits in leaves, culms, rhizomes, and shoots, respectively, with 10 *PeAAP*s identified among the coexpression network (Fig. S6).

**Figure 4:**
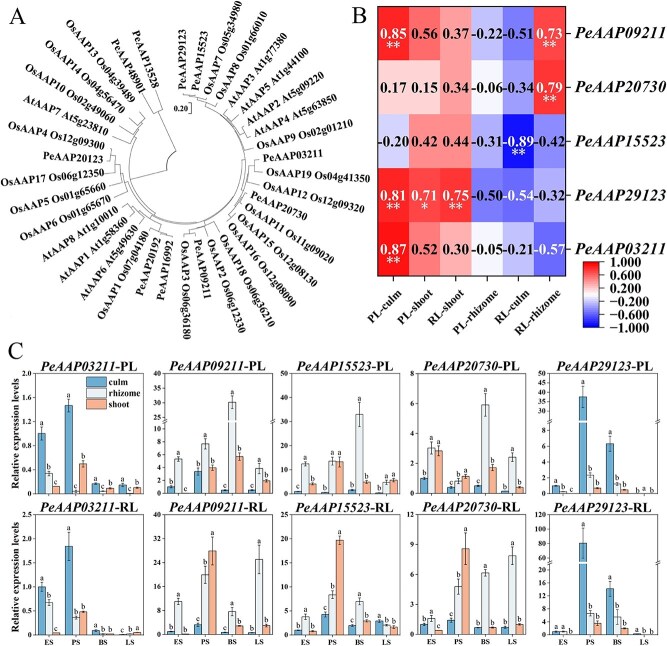
Expression analysis of *PeAAP*s in Moso bamboo under ^15^N labelling treatments. (A) Evolutionary tree of AAPs from Moso bamboo, *Arabidopsis thaliana,* and rice. (B) Heat map depicting correlations between the expression of *PeAAP*s and the ^15^N content in different organs. The asterisks indicate significant correlation (^*^P < 0.05, ^**^P < 0.01). (C) RT-qPCR results of five *PeAAP*s in different organs within clonal fragment under ^15^N labelling treatments. PL and RL represent parent ramet labelling and rhizome rhizosphere labelling treatments, respectively. ES, PS, BS, and LS represent the early, peak, branching, and leafing stage of rapid growth of offspring ramets, respectively. Data are shown as mean ± standard deviation (*n* = 3). Different lowercase letters indicate significant differences among organs during each stage. Culm belong to parent ramet, and shoot belong to offspring ramet.

The function of AAP homologous genes has been previously verified in other species [27]. We further analysed the evolutionary relationships of the AAPs in Moso bamboo, Arabidopsis, and rice. *PeAAPs* were more closely related to *OsAAPs* (rice) than to Arabidopsis, suggesting that they may have similar functions (Fig. 4A). Pearson correlation analysis of the relative expression levels of five genes and ^15^N content revealed that distinct *PeAAP*s exhibited organ-specific transport of ^15^N; among them, *PeAAP29123* facilitated ^15^N transport in both culm and shoot (Fig. 4B). We further analysed the expression levels of five highly expressed *PeAAP*s in the samples of ^15^N labelling treatments by using RT-qPCR. The results showed that *PeAAP29123* and *PeAAP03211* were highly expressed in the rhizome and culm of parent ramet, whereas *PeAAP09211*, *PeAAP15523*, and *PeAAP20730* were highly expressed in rhizomes and shoots (Fig. 4C).

### Heterologous overexpression of *PeAAP29123* in rice

**Figure 5:**
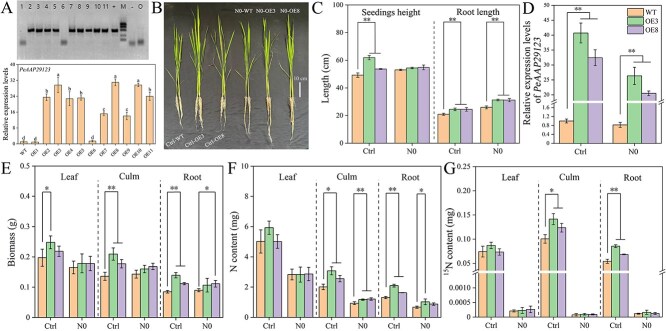
Overexpression of *PeAAP29123-*accelerated N uptake in transgenic rice. (A) Positive plant detection. (B) Phenotype of WT and *PeAAP29123* transgenic lines in rice (OE3, OE8) in control (Ctrl) and N starvation (N0) treatments. (C) Seedling height and root length of WT and *PeAAP29123* transgenic rice lines in control and N starvation treatments. (D) Relative expression of *PeAAP29123* gene in leaves of rice under control and N starvation treatments. (E, F, G) The biomass, N content, and ^15^N content in different organs of WT and *PeAAP29123* transgenic rice lines in control and N starvation treatments. Data are shown as mean ± standard deviation (*n* = 3). The asterisk indicates a significant difference between WT rice and *PeAAP29123* overexpressing rice (^*^P < 0.05, ^**^P < 0.01).

To clarify the role of *PeAAP29123* in N transport, the function of *PeAAP29123* was examined by heterologous transformation into rice. Indeed, PCR and RT-qPCR results showed that nine positive strains were obtained, among which, the transcription levels of strains PeAAP29123_OE3 and PeAAP29123_OE8 were significantly higher than those of other strains. The positive seedlings of these two strains were selected for subsequent experiments. Results showed that seedling height, root length, biomass, and N and ^15^N content of culms and roots in *PeAAP29123* transgenic lines were significantly higher in the control treatment than those of wild-type (WT) plants. Conversely, under N starvation (N0), the biomass, N and ^15^N content of both WT and *PeAAP29123* transgenic lines decreased but root length increased compared to that under control treatment (Fig. 5). These results indicated that the overexpression of *PeAAP29123* promoted biomass accumulation and N uptake in the transgenic rice lines.

### Transcriptional regulation of *PeAAP29123* by PeHDZ23987

To identify potential transcription factors (TFs) involved in the transcriptional regulation of N transporter-protein-related genes during the rapid growth of offspring ramet, the correlations between TFs and genes encoding for amino acid-transporter proteins were analysed. Thus, we used 2000 bp upstream sequences in the promoter of *PeAAP29123* to predict *cis*-regulatory elements in the PlantRegMap database (Table S5). Several important *cis*-elements of TFs (such as bZIP, C2H2, Dof, ERF, HD-ZIP, and MYB) were identified (Table S6). Meanwhile, among the 1562 TFs, 28 were identified as coexpressed and significantly correlated with genes encoding amino acid-transport proteins (Table S7 and S8). The identification of these binding sites in 20 out of the 28 TFs offered valuable insights into the transcriptional regulation mechanisms of Moso bamboo N transporter-protein-related genes. G2-like (*PeGLK09101*) and HD-ZIP (*PeHDZ23987*) were highly correlated with *PeAAP29123* (r = 0.789 and r = 0.648, respectively). Therefore, we speculated that PeGLK09101 and PeHDZ23987 probably regulate the expression of *PeAAP29123*.

**Figure 6:**
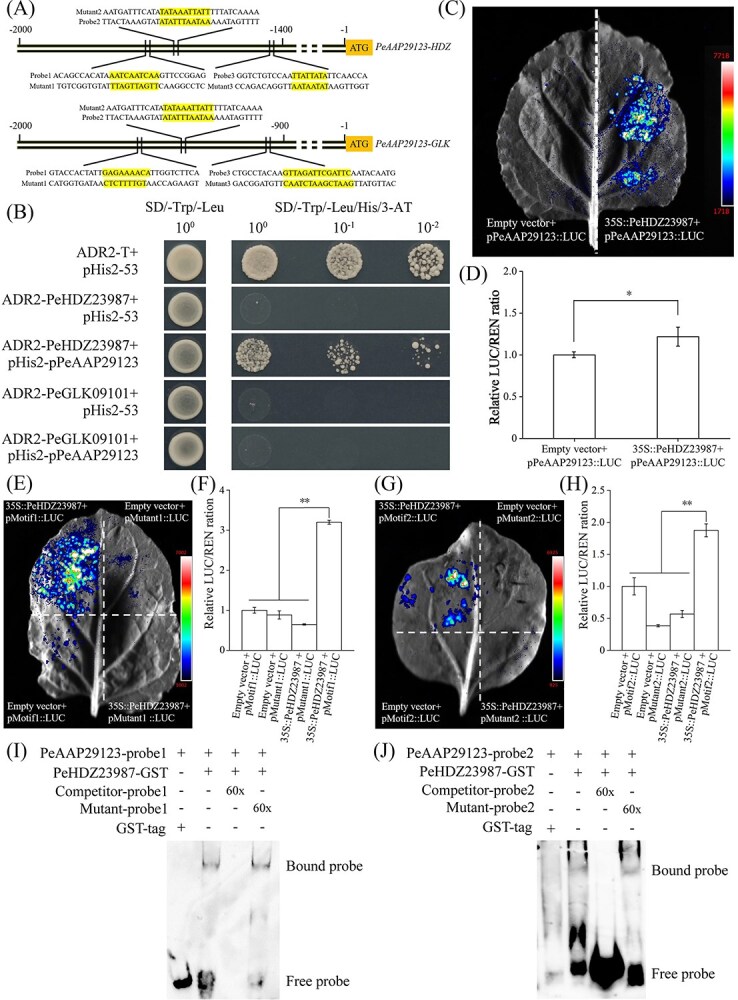
PeHDZ23987 activated *PeAAP29123* by binding to its promoter. (A) The distribution diagram of TF binding sites of proPeAAP29123. (B) Y1H assays were used to identify PeHDZ23987 binding to the promoter of *PeAAP29123*. (C) Dual-luciferase complementation imaging assays used to show that PeHDZ23987 activated the expression of *PeAAP29123*. (D) The firefly luciferase (LUC)/Renilla luciferase (REN) (LUC/REN) activity ratio was used to verify that PeHDZ23987 activated *PeAAP29123*. (E, G) PeHDZ23987 activating the transcription of HD-motifs of *PeAAP29123* in tobacco leaves by dual-luciferase assays. pMotif1, pMotif2, pMutant1, and pMutant2 indicated pPeAAP29123-Motif1::LUC, pPeAAP29123-Motif2::LUC, pPeAAP29123-Mutant2::LUC, and pPeAAP29123-Mutant1::LUC, respectively. Representative photographs were taken using a chemiluminescence imaging system. (F, H) LUC/REN activity detection to verify PeHDZ23987 activating the transcription of HD-motifs of *PeAAP29123*. Data are shown as mean ± standard deviation (*n* ≥ 3). The asterisk indicates a significant difference (^*^P < 0.05, ^**^P < 0.01). (I–J) PeHDZ23987 binds to two motifs (HDZ-motif1 5'-CAATCAAG-3′ and HDZ-motif2 5'-CAATTATT-3′) of the *PeAAP29123* promoter by EMSA. Unlabelled probes were used as competitors. The 60× represent the concentrations of the competitor. Mut represents a mutated probe in which the motif is replaced by 5'-ACGGCTCC-3′.

The regulatory relationships among PeGLK09101, PeHDZ23987, and *PeAAP29123* were analysed by yeast one-hybrid (Y1H) assay. All yeast cells grew in the SD/−Leu/−Trp (DDO) medium, while only the yeast cells coexpressing ADR2-PeHDZ23987 and pHIS2-pPeAAP29123 grew normally in SD/−Leu/-His/−Trp + 50 mM 3-AT (TDO + 3-AT) medium. These results suggested that PeHDZ23987 effectively bound to the promoter of *PeAAP29123*, whereas PeGLK09101 did not (Fig. 6B). To further determine whether PeHDZ23987 directly regulates *PeAAP29123*, dual-luciferase assays were conducted. The fluorescence signals of the cotransformed 35S-PeHDZ23987 + pPeAAP29123-LUC regions are stronger than those of SK + pPeAAP29123-LUC regions (Fig. 6C) The firefly luciferase (LUC)/Renilla luciferase (REN) activity ratio of 35S-PeHDZ23987 + pPeAAP29123-LUC was significantly higher than that of the empty vector (Fig. 6D). These results confirmed that PeHDZ23987 was able to activate the expression of *PeAAP29123*. Furthermore, the *in vivo* physical interaction between PeHDZ23987 and two HD-motifs was analysed using dual-luciferase reporter (DLR) assay. Cotransformed 35S::PeHDZ23987 with pMutant::LUC, and SK with pMutant::LUC were used as negative controls. LUC luminescence and activity were observed when pMotif1::LUC and PeHDZ23987, pMotif2::LUC, and PeHDZ23987 were cotransformed into tobacco leaves, which showed higher luminescence than the negative controls (Fig. 6E–H).

Additionally, electrophoretic mobility shift assay (EMSA) demonstrated the direct interaction between PeHDZ23987 and *PeAAP29123* promoter. PeHDZ23987-GST fusion proteins exhibited a strong tendency to bind to the *PeAAP29123* probes, and the binding was weakened in the presence of a competitor probe (Fig. 6I and J). Furthermore, the PeHDZ23987_KD was generated by using the *in planta* gene editing method (Fig. S7A–C) to explore the expression dynamics of PeHDZ23987 and *PeAAP29123*. The results showed that the knockdown of *PeHDZ23987* inversely led to decreased relative expression of *PeHDZ23987* and three *PeAAP*s, compared with CK (Fig. S7D–I), suggesting that PeHDZ23987 might regulate the expression of *PeAAP29123* in Moso bamboo. Altogether, these data indicate that PeHDZ23987 may play a role in N transport by interacting with specific binding sites of the *PeAAP29123* promoter in an HD-motif-dependent manner.

### Heterologous overexpression of *PeHDZ23987* in rice

**Figure 7:**
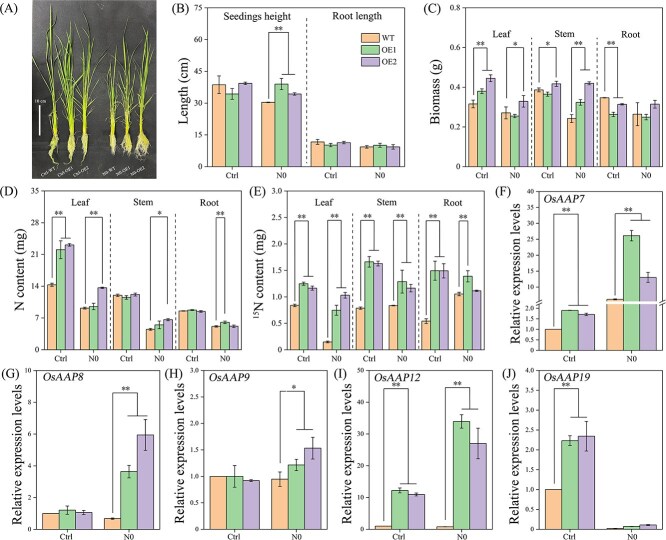
Overexpression of *PeHDZ23987-*accelerated N uptake in transgenic rice. (A) Phenotype of WT and *PeHDZ23987* transgenic lines in rice (OE1, OE2) in control (Ctrl) and N starvation (N0) treatments. (B) Seedling height and root length of WT and *PeHDZ23987* transgenic rice lines in control and N starvation treatments. (C–E) The biomass, N content, and ^15^N content in different organs of WT and *PeHDZ23987* transgenic rice lines in control and N starvation treatments. (F–J) Relative expression of *OsAAP7*, *OsAAP8*, *OsAAP9*, *OsAAP12*, and *OsAAP19* genes in leaves of rice under control and N starvation treatments. Data are shown as mean ± standard deviation (*n* = 3). The asterisk indicates a significant difference between WT rice and *PeHDZ23987* overexpressing rice (^*^P < 0.05, ^**^P < 0.01).

To further investigate the role of PeHDZ23987 in N transport, we heterologously expressed *PeHDZ23987* in rice. Positive seedlings of two strains (PeHDZ23987-OE1 and PeHDZ23987-OE2) were selected for N starvation stress experiments. The results showed that plant height and N and ^15^N content were significantly higher in the N starvation (N0) treatment than those in the WT plants. Biomass and ^15^N content of the *PeHDZ23987* transgenic lines were significantly higher in the control treatment than those in the WT plants (Fig. 7A–E). Additionally, the biomass and N and ^15^N content in leaves of both WT and *PeHDZ23987* transgenic lines under N0 treatment were reduced compared to those in the control (Fig. 7C–E). Furthermore, four *OsAAP*s (*OsAAP7*, *OsAAP8*, *OsAAP9,* and *OsAAP12*) were upregulated in both the PeHDZ23987-OEs under N starvation stress, in comparison to the WT (Fig. 7F–J). These results indicate that the overexpression of *PeHDZ23987* promotes biomass accumulation and N uptake by promoting the transcription of *OsAAP*s in the transgenic rice seedlings.

## Discussion

### Dynamic N supply during rapid growth of Moso bamboo

Parent ramet and rhizome roots showed an obvious ‘suckle’ behaviour during the rapid growth of offspring ramet, indicating that clonal integration plays a critical role in the nutrient transport for the growth of offspring ramet when nutrients are unbalanced between the parent and offspring ramets [13, 25]. Culms of the parent ramet are known to provide large amounts of N to support the rapid growth of offspring ramet, due to their substantial nutrient storage capacity (Fig. S1; [14]). Moreover, high *PeAAP* expression in culms of the parent ramets during the peak stage also revealed increased N transport activity (Fig. 3D). Interestingly, when rhizome rhizosphere was labelled, the bilateral supply of ^15^N was noted and the proportion supplied to each (parent or offspring ramet) was demand-driven. During peak stage, the unequal supply pattern indicated ^15^N allocation was sink strength-dependent. Thus, as an N source, rhizome roots may act as regulators, balancing the N concentration difference between clonal ramets. Additionally, a ^15^N backflow phenomenon observed during the leafing stage, particularly when the parent ramet was labelled, may suggest a reversal in the direction of clonal integration. This phenomenon is closely associated with the developmental stage [28]. Previous studies suggest that these resource transfer reversals are reasonable at later stages of offspring ramet development [29, 30]. The translocation of resources between ramets is often reciprocal; offspring ramets may support their parent ramets but the amounts of nutrients that they can allocate to their parent ramets are either much smaller [31] or about the same [32], compared to the amounts they received. Compared to rhizome labelling, the higher backflow and accumulation of ^15^N in parent leaves observed with parent ramet labelling indicate that N supply from the parent ramet may be phased, dynamic, and demand-driven, and can be compensated by the N supply from the rhizome roots.

### Modified N acquisition strategy of offspring ramet during rapid growth

The contributions of parent ramet and rhizome roots to the rapid growth of offspring ramet varied across different growth stages, reflecting a shift in the N acquisition strategy from being primarily supplied by the parent ramet to predominantly being absorbed by the rhizome roots. This transition underscores the division of labour between parent ramet and rhizome roots in providing N to the offspring ramet. Meanwhile, changes in the cost of N acquisition may be one of the reasons for offspring ramet to adjust their N acquisition strategies. The parent ramet supplied a significant amount of N during the early stage, when the N demand of offspring ramet was relatively low and N transport from parent to offspring ramet was energetically free or low-cost [29]. During the peak and branching stages, the allometric growth of offspring ramet required larger amounts of N compared with that during early stage, such that the parent ramet and rhizome root worked synergistically, and contributed almost equally in supplying N to the offspring ramet. As the TN content of the parent ramet decreased (Fig. S1), the cost of N transport from parent ramet to the offspring ramet increased [29], whereby offspring ramet may incur higher costs to obtain N from the parent ramet relative to N absorption from rhizome roots. Therefore, the N supply from the parent ramet decreased while concomitantly, the rhizome root contributed most of the absorbed N to the growth of offspring ramet during the leafing stage. Meanwhile, this change in the N acquisition strategy of offspring ramet avoided lowering fitness of the parent ramets [33, 34]. It must be acknowledged that the results of our study were obtained without considering the N uptake by the roots of offspring ramet during the rapid growth, as they remained undeveloped roots at this stage. However, as the root system continued to develop, we can speculate that the N acquisition strategy of offspring ramet would continue to change, shifting towards a more significant contribution from self-root absorption for their later growth.

### *PeAAP*s play an important role in N transport between clonal ramets

Amino acid permeases have been reported to localize in the phloem of Arabidopsis, pea, and common beans (*Phaseolus vulgaris*), where they are presumed to facilitate amino acid transport from the apoplast into the phloem [35–38]. In this study, *PeAAP29123* was highly expressed in culms of the parent ramets and shoots (culms of offspring ramet) during the peak and branching stages in the control treatment. Further, *PeAAP29123* expression significantly increased in the culms and rhizomes within the clonal fragments during both stages in the ^15^N labelling treatments. We hypothesize that *PeAAP29123* is involved in long-distance N transport between source and sink organs in Moso bamboo. Additionally, the overexpression of *GmAAP6a* reportedly enhances tolerance to low N and improves seed N status by optimizing amino acid partitioning in soybean [25]. *SlAAP6* has been reported to contribute to growth and salt tolerance in tomato by mediating branched-chain amino acid transport [27]. In this study, the expression pattern of *PeAAP29123* was positively correlated with ^15^N content in the culms of parent ramets and shoots when the former were labelled. Furthermore, overexpression of *PeAAP29123* improved seedling biomass and N status in transgenic rice, indicating that it serves as a positive regulator of N transport. These results indicate that *PeAAP29123* played an important role in regulating N transport from the culms of parent ramets to the offspring ramets. Although AAPs have overlapping transporter substrates, they have different expression patterns and specialized functions [39]. The correlation between the expression levels of other *PeAAP*s and the content of ^15^N in different organs was different from that of *PeAAP29123* (Fig. 4C), indicating that N transport from parent to offspring ramet was mediated by various *PeAAP*s; moreover, different *PeAAP*s exhibited organ-specific N transport.

### The PeHDZ23987–*PeAAP29123* module is involved in N supply of bamboo shoots

TFs are key regulators of gene expression and play important roles in regulating plant growth and development. In particular, HD-ZIP proteins are plant-specific TFs characterized by one DNA-binding domain, one zipper motif, and one homeodomain. A growing body of evidence suggests that HD-ZIP TFs are involved in the regulation of plant architecture, organogenesis, and reproductive processes [40–42]. For example, *ATHB1* plays a crucial role in developmental processes in tobacco (*Nicotiana tabacum*) leaf cells [43]. Similarly, transgenic plants overexpressing *ATHB23* or *ATHB3*, *ATHB13,* and *ATHB20* significantly fine-tuned cotyledon and leaf developmental processes [44, 45]. A recent study has shown that PeHDZ72 participated in water and sugar transport processes by increasing the expression of downstream genes [46]. In this study, an HD-ZIP member (*PeHDZ23987*) was identified in Moso bamboo. We observed that *PeHDZ23987* was coexpressed with *PeAAP29123* in shoots during the rapid-growth stages. Additionally, DLR and EMSA assays indicated that PeHDZ23987 activated the transcription of *PeAAP29123*. Moreover, overexpression of *PeHDZ23987* significantly improved N-transport capacity of transgenic rice lines; *OsAAP7* and *OsAAP8* that act as general amino acid osmotic-agents, were markedly upregulated in PeHDZ23987_OEs under N starvation stress [21]. Additionally, HD-motifs are considered to be a typical binding element of HD-Zip TFs in plants [46, 47]. In this study, two HD-motifs (pMotif1: CAATTATT and pMotif2: CAATCATT) were verified to be bound by PeHDZ23987. Therefore, we inferred that PeHDZ23987 may participate in the N-supply process by activating the expression of *PeAAP29123* through specific elements. Based on our findings and previous studies, we propose that PeHDZ23987 improves the efficiency of amino acid transport by promoting the expression of *PeAAP29123.*

## Conclusions

The results presented here demonstrate for the first time that the division of labour and cooperation between parent ramet and rhizome roots for N supply jointly supported the rapid growth of Moso bamboo offspring ramet. During the early stage, the offspring ramets primarily relied on N provided by the parent ramet; however, this shifted predominantly to N absorbed by rhizome roots by the leafing stage. The N-transporter-related gene, *PeAAP29123,* plays a critical role in supplying N to offspring ramet under regulation by TF, PeHDZ23987. Overall, our findings reveal the mechanism responsible for nutrient supply in offspring ramets of Moso bamboo during their rapid growth stage and provide transcriptomic evidence of long-distance amino acid transport between clonal ramets. These insights offer a theoretical basis for rational fertilization of horticultural plants represented by clonal plants of bamboo.

## Materials and methods

### Site description

The experiments were conducted in Lin’an, Hangzhou, China (30.24°N, 119.42°E). The study site is characterized by a subtropical monsoon climate with a mean annual temperature of 15.6°C, a mean annual precipitation of 1420 mm, and a 230-day frost-free period. The study area receives 1847 h of sunlight per year. A Moso bamboo forest was established in this area in the late 1970s [48]. Annual harvesting of bamboo shoots (offspring ramet) and biennial thinning of mature bamboo were conducted, creating an alternating production pattern of ‘on-years’ and ‘off-years’. The stand density in this forest is 3362 ± 309 culms ha^−1^, consisting solely of 2- and 4-year-old culms.

### Experimental design and sample collection

To explore the N supply process during the rapid growth of Moso bamboo offspring ramets, 36 pairs of clonal fragments composed of a parent ramet (2-year-old bamboo), an offspring ramet, and an interconnecting rhizome were constructed in the Moso bamboo forest (Fig. S3a). Briefly, a healthy offspring ramet with a height of 20 ± 2 cm and a ground diameter of 8 ± 1 cm was randomly selected, and the soil around the offspring ramet was slightly removed to locate the connected rhizome. Then the parent ramet connected with the offspring ramet was eventually found along with the opposite direction of rhizome elongation, and the rhizomes out of the clonal fragment were excised.

The experiment included three treatments, ^15^N labelling in the parent ramet (PL), ^15^N labelling in the rhizome rhizosphere (RL), and the control without ^15^N labelling. Three plots (30 × 30 m) were established, each including 12 pairs of clonal fragments corresponding to the three treatments during four growth stages. On 2 April 2021, 10 ml of ^15^NH_4_^15^NO_3_ solution with a concentration of 50 mg N ml^−1^ (30.14 atom%; Shanghai Engineering Research Centre of Stable Isotopes, China) were applied to parent ramets by cavity injection [13] and to the rhizosphere soil of the rhizome.

On 7, 21, 34, and 81 days after ^15^N labelling, corresponding to early (ES), peak (PS), branching (BS), and leafing (LS) stages of rapid growth of offspring ramet based on growth rate and anatomical characteristics (Fig. S3b, [7, 8]), the leaves, culm of parent ramet (including branches, aboveground and belowground culms), rhizome, shoot (culm), and nascent leaf of offspring ramet were collected between 8 and 11 a.m. For control, transcriptome sequencing samples were collected from leaves (mixture of upper, middle, and lower leaves in the crown), culms (a long strip of the 13th bamboo node), rhizomes (the middle part), and shoots (same as culms) of the clonal fragment. These samples were washed three times with sterile water, then, dried using absorbent paper, flash-frozen in liquid N, and immediately stored at −80°C upon returning to the laboratory. The remaining organs were dried to a constant mass and weighed to calculate water content and biomass. Dried samples were ground and passed through a 0.15-mm sieve in preparation for the N content and ^15^N abundance determinations.

### Measurement of indexes and calculations

TN concentration and atom% ^15^N in different organs were determined using EA-IRMS (Thermo Fisher DELTA V Advantage, USA). The concentrations of NO_3_^−^—N, NH_4_^+^—N, and soluble protein in each sample were determined using nitrosalicylic acid colorimetry, ninhydrin colorimetry, and the Coomassie brilliant-blue method, respectively, according to manufacturer instructions (Suzhou Comin Biotechnology Co., Ltd., China).

The percentage of ^15^N derived from ^15^NH_4_^15^NO_3_ (Ndff, %) was calculated using equation (1) [50]:


(1) \begin{equation*} Ndff\left(\%\right)=\frac{b-a}{c-a}\times 100 \end{equation*}

where a is the atom% ^15^N in the organs of the control, b is the atom% ^15^N in the organs of the ^15^N-labelling treatments, and c is the atom% ^15^N in the fertilizer (30.14 atom%).

The ^15^N concentration and content in each organ were calculated as follows [13]:


(2) \begin{equation*} {}{}^{15}{N}_{organ}\ \left( mg\ {kg}^{-1}\right)={N}_{organ}\ \left( mg\ k{g}^{-1}\right)\times Ndf{f}_{organ}\times 1{0}^{-2} \end{equation*}

(3) \begin{equation*} {}{}^{15}{N}_{organ}\ (g)={{}{}^{15}N}_{organ}\ \left( mg\ k{g}^{-1}\right)\times{B}_{organ}\ (kg)\times 1{0}^{-3} \end{equation*}

where N_organ_ is the N concentration in each organ, B_organ_ is the biomass of each organ.

^15^N recovery was calculated using equation (4):


(4) \begin{align*} {}{}^{15}N\ &recovery\ \left(\%\right)\nonumber\\&=\frac{\ {}{}^{15}{N}_{leaf}+{}{}^{15}{N}_{culm}+{}{}^{15}{N}_{rhizome}+{}{}^{15}{N}_{shoot}+{}{}^{15}{N}_{nascent\ leaf}}{labelled\ {}{}^{15}N}\times 100\% \end{align*}

where ^15^N_leaf_, ^15^N_culm_, ^15^N_rhizome_, ^15^N_shoot_, and ^15^N_nascent leaves_ represent ^15^N content (g) in leaf, culm, rhizome, shoot, and nascent leaf, respectively. ^15^N_nascent leaves_ only appeared at the leafing stage. Labelled ^15^N was 150.7 mg.

### Transcriptome sequencing analysis

Total RNA extraction from different bamboo organs (4 growth stages × 4 organs × 3 biological replicates) was performed according to RNA extraction kit (Takara Bio, Inc., Otsu, Japan). The RNA quality was detected by 1% agarose gel electrophoresis and a NanoDrop ND-2000 spectrophotometer [51]. The RNA concentration and integrity were determined by Qubit 2.0 and Agilent 2100 Bioanalyzer System (Agilent Technologies, Santa Clara, CA, USA), respectively. Then, RNA was sequenced by transcriptomic RNA-Seq to construct an RNA-Seq cDNA library [52]. Finally, the library was sequenced on the Illumina Novaseq platform. After data disembarkation, the filtering software SOAPnuke was used to filter the sequencing data.

Sequencing data were mapped to the Moso bamboo genome using HISAT 2 [53]. Gene expression levels were estimated as fragments per kilobase of transcript per million fragments mapped (FPKM) [54]. The DEGs analysis was performed using DESeq2 [55]. A *P-*value <0.05 and |log_2_Fold Change| ≥1 were set as thresholds for defining significant differential gene expression.

### Quantitative real-time PCR

Primers were designed using Primer Premier 5 software (PREMIER Biosoft, CA, USA) (Table S9). First-strand cDNA synthesis was performed using a reverse transcription kit (Takara Bio, Inc.) according to the manufacturer’s instructions. The RT-qPCR reactions were performed with the SYBR® Premix Ex Taq™ (Tli RNaseH Plus) qPCR Kit (Takara Bio, Dalian, China) using Bio-Rad CFX96 (Bio-Rad Laboratories, Hercules, CA, USA) as previously described [19], with *PeNTB* as the endogenous control gene [56]. Three technical and biological replicates were included for each experiment. Gene expression levels were calculated using the 2^-ΔΔCT^ method [57].

### Yeast one-hybrid assay and dual-luciferase assay

Y1H assay: The open reading frame (ORF) sequences of *PeGLK09101* and *PeHDZ23987* were cloned into the pGADT7-Rec2 vector (ADR2-PeGLK09101 and ADR2-PeHDZ23987). The promoter sequence of *PeAAP29123* was inserted into the pHIS2 vector (pHIS2-pPeAAP29123). Different combinations were cotransformed into yeast Y187, and the interactions were tested in a TDO medium with an optimal concentration of 3-amino-1,2,4-triazole (3-AT).

Dual-luciferase assay: The ORF sequence of *PeHDZ23987* gene was inserted into the pGreenII 62-SK vector as effector (35S-PeHDZ23987). The *PeAAP29123* promoter fragment as well as the *cis*-elements and mutant *cis*-element parts were inserted into the pGreenII 0800-LUC vector upstream of the LUC gene used as reporters (pPeAAP29123::LUC, pMotif1::LUC, pMutant1::LUC, pMotif2::LUC, and pMutant2::LUC). The recombinant vectors were transformed into *Agrobacterium* strain GV3101 (pSoup). Tobacco leaves were infected with a mixture of *Agrobacterium* strains. After 3 days of infiltration, the LUC fluorescence signal was detected using a chemiluminescence imaging system (Tiano4800, China), and LUC and REN luciferase activities were measured using the dual-luciferase® reporter assay system (Vazyme, DL101–01, China) with a single-tube luminescent detector (Lumipro, China).

### Electrophoretic mobility shift assay

EMSA was performed using a chemiluminescent EMSA kit (Beyotime, GS009, China) according to manufacturer's instructions. The sequences encoding full-length of PeHDZ23987 were cloned into pGEX4T-1 vector harbouring an N-terminal GST tag. The GST::PeHDZ23987 fusion proteins were produced in *Escherichia coli* strain BL21 (DE3) with 0.2 mM sopropyl-β-α-1-thiogalactopyranoside for 16 h at 15°C. The probes containing HDZ-motif from the *PeAAP29123* promoter were labelled with biotin on 3′ ends. The same unlabelled probes were used as a cold competitor. EMSA was performed by incubating the probes with GST::PeHDZ23987 at 25°C for 1 h. The reaction products were analysed using 6% native polyacrylamide gel electrophoresis in 0.5× Tris/Borate/EDTA buffer. The chemiluminescence signal was detected using a chemiluminescence imaging system (Tanon 4800, China).

### *In situ* gene editing in Moso bamboo leaves

The leaves of 2-month-old Moso bamboo seedlings were utilized for the *Agrobacterium*-mediated *in planta* gene editing. The sgRNA leader sequence for PeHDZ23987 was designed and inserted into the pCAMBIA1300-Ubi::Cas9 vector, and then transformed into *Agrobacterium tumefaciens* strain GV3101. The pCAMBIA1300-Ubi::Cas9 empty vector and transformant of pHDE-35S::RUBY were used as the control (CK) and positive visual indicator, respectively [58]. *Agrobacterium* strains carrying PeHDZ23987 CRISPR/Cas9 vector or CRISPR/Cas9 vector and RUBY reporter gene construct were combined in a 3:1 ratio. Subsequently, the bamboo leaves were infected using the procedure outlined in a previous report [58]. The results of gene editing were detected by PCR amplification with product sequencing and digestion to determine the mutant types, and RT-qPCR to examine the relative expression levels of *PeHDZ23987* and *PeAAP*s using gDNA and total RNA isolated as described before.

### Generation of transgenic plant materials and N starvation treatment

The ORF sequences of *PeAAP29123* and *PeHDZ23987* were inserted into the expression vector pCAMBIA1300 with a Ubi promoter, according to the manufacturer instructions provided with the PCR Cloning Kit (Novoprotein, NR005-01B, China). The resulting plasmids were introduced into the *Agrobacterium* strain GV3101 (Zomanbio, ZC141, China) using the freeze–thaw method. The transformed strain was used to transform rice (*Oryza sativa* ‘Nipponbare’) calli, which were inoculated on a screening medium and incubated at 26°C for 20–30 days [49]. The positive calli were inoculated into a secondary screening medium and cultured in the dark at 26°C for 7–10 days. The positive calli were inoculated into the differential medium and cultured under light at 25°C–27°C for 15–20 days. After 2- to 5-cm buds were differentiated, they were inoculated into the rooting medium and cultured at 30°C for 7–10 days under light. Rice genomic DNA was extracted using the CTAB method and tested using PCR.

Rice seedlings were cultivated in an optimal environment with a photoperiod of 12 h light and 12 h dark, a light intensity of ~300 μmol·m^−2^·s^−1^, a temperature ranging from 26°C to 30°C, and an ambient relative humidity of ~60% [59]. Hydroponic experiments included control and N starvation treatments, with three replicates per treatment. WT and transgenic rice seedlings with a plant height of 40 cm and uniform growth were transplanted into 3-l buckets. Kimura B solution was used in the control group, and N-free Kimura B solution was used in the N starvation treatment group (see Table S10 for the full formula of the hydroponic solution). On the fifth day of the experiment, the nutrient solution in the control group was adjusted to be half Kimura B solution and half N-free Kimura B solution, and 41.82 mg of ^15^NH_4_^15^NO_3_ was added to maintain the N concentration of the solution at 10.2 mg N·l^−1^). Plant height, root length, biomass, TN content, and ^15^N abundance were determined on the seventh day of the experiment.

### Statistical analysis

The data used for statistical analysis fit a normal distribution; otherwise, they were square root transformed. One-way analysis of variance (ANOVA), student’s *t*-test, and Duncan’s multiple comparison tests were performed using SPSS 20.0. Graphs were constructed using Origin 2023b software. MEGA11 was applied to compare multiple sequences of *PeAAP*s, *AtAAP*s, and *OsAAP*s. A phylogenetic tree was constructed using the neighbour-joining method, and 1000 bootstrap replications were performed. Cytoscape (version 3.10.1) was used to visualize gene–trait interaction networks.

## Supplementary Material

Web_Material_uhaf062

## Data Availability

Isotope data will be made available on request. The RNA-Seq data has been deposited to the NCBI SRA database under the BioProject PRJNA1085513.
