# Supplementary material for: Stable isotope labelling and gene expression analysis reveal dynamic nitrogen-supply mechanisms for rapid growth of Moso bamboo
Source: Hortic Res. 2025 Feb 25;12(6):uhaf062. doi: 10.1093/hr/uhaf062 (PMC12023858; doi:10.1093/hr/uhaf062)
Supplement: Web_Material_uhaf062 [file web_material_uhaf062.zip › Supplementary Figures S1 - S7.docx]

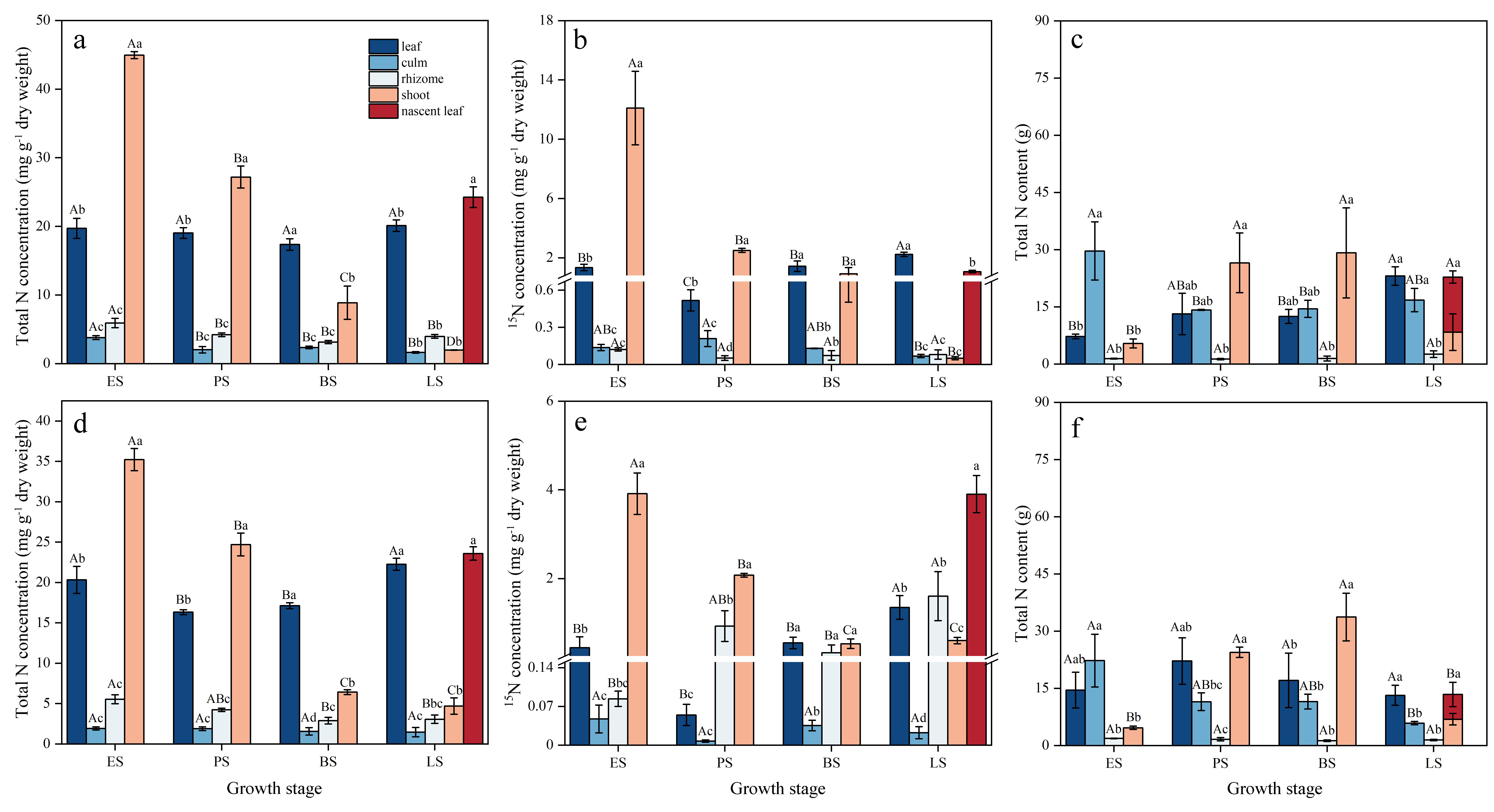


**Fig. S1 The changes of total N and ^15^N concentration and total N content in different organs within clonal fragment during different growth stages when parent ramet (a, b, c) and rhizome rhizosphere (d, e, f) were labeled with ^15^N.** ES, PS, BS, and LS represent the early stage, peak stage, branching stage, and leafing stage of rapid growth of Moso bamboo offspring. Different capital letters indicate significant differences among different stages in each organ. Different lowercase letters indicate significant differences among different organs during each stage.


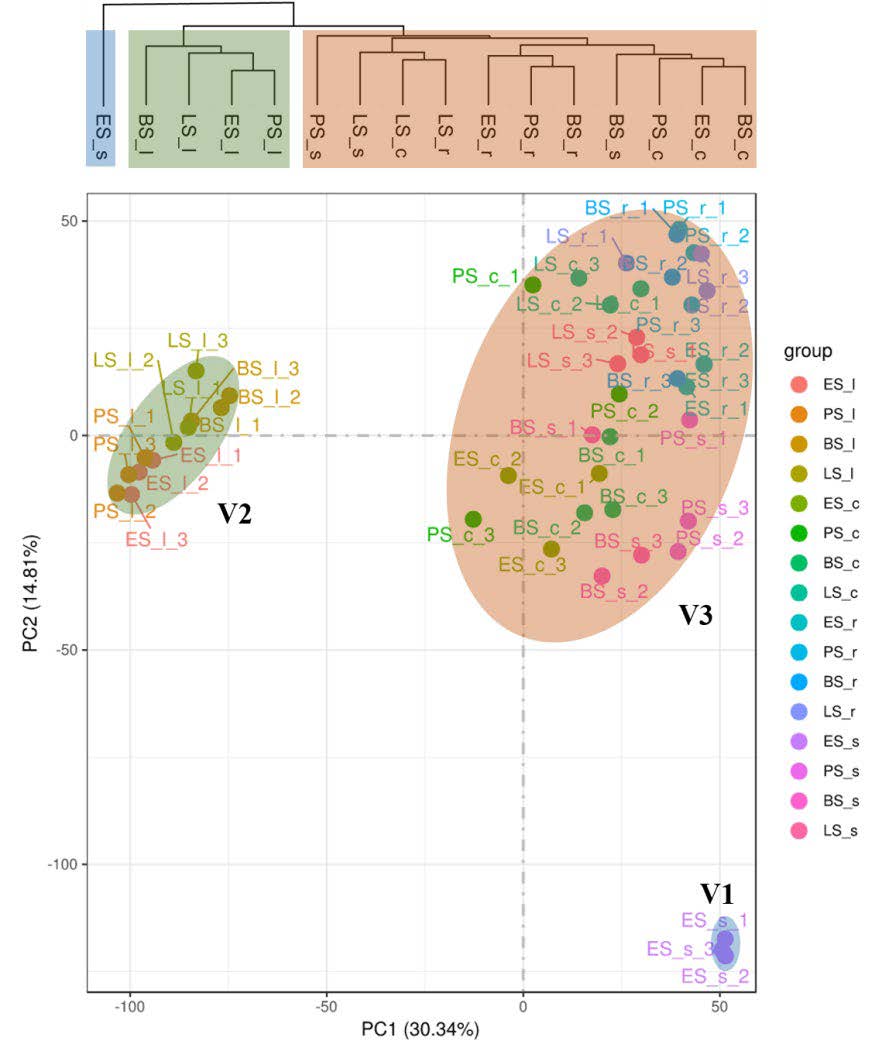


**Fig. S2 Cluster tree and principal component analysis of the transcriptome of 16 groups of samples**


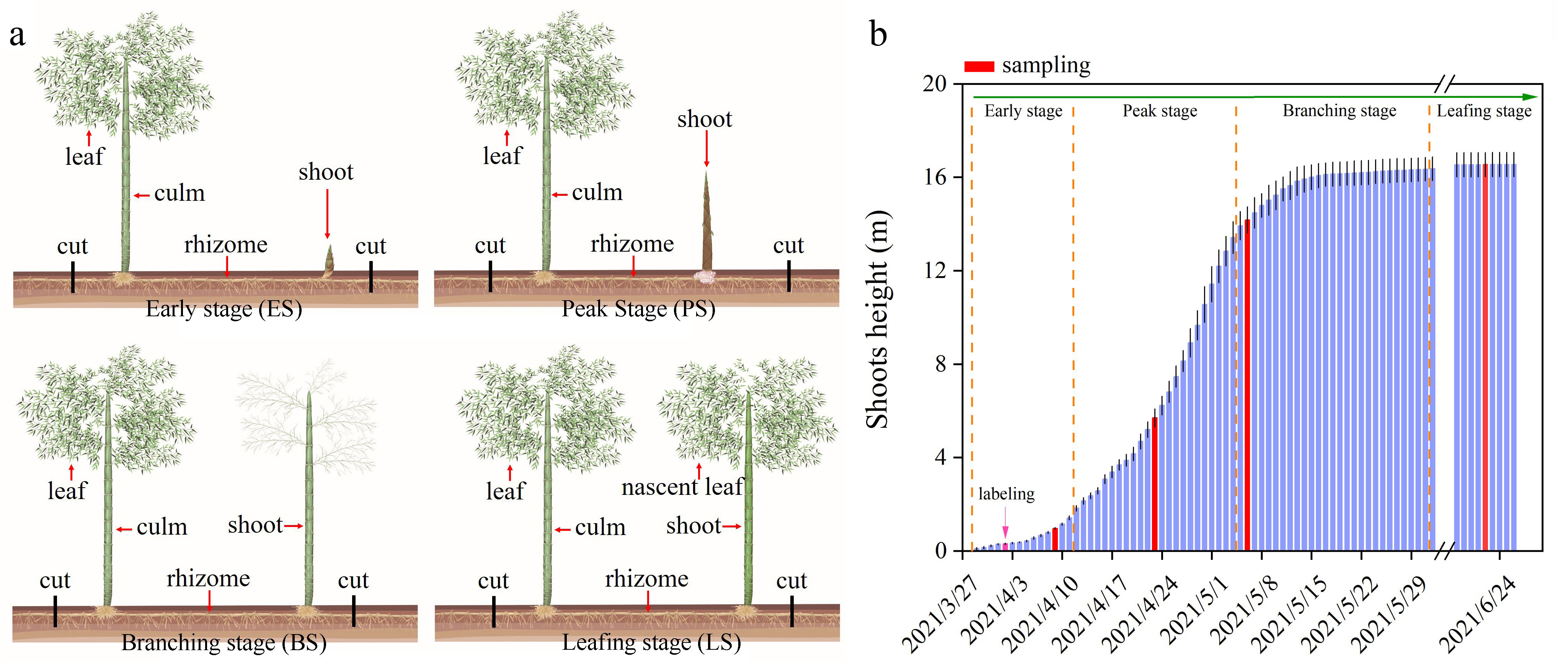


**Fig. S3 Schematic diagram of clonal fragments during different growth stages of Moso bamboo shoot.** The pink bar represents the time of ^15^N labelling; The red bars represent the time of sampling.


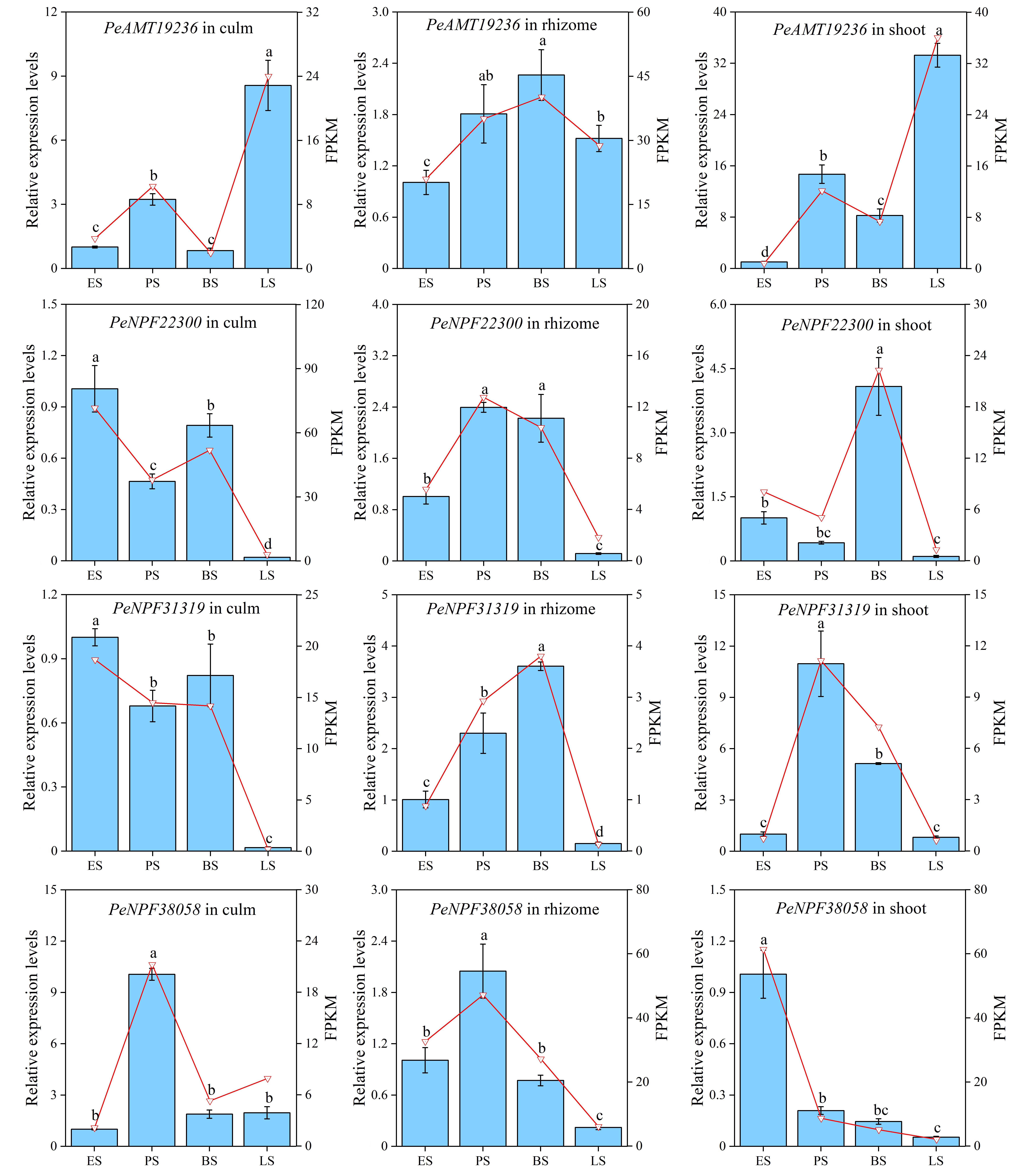


**Fig. S4 RNA-Seq (broke line) and qPCR (bar chart) data for six genes in three different organs.** ES, PS, BS, and LS represent the early stage, peak stage, branching stage, and leafing stage of rapid growth of Moso bamboo offspring.


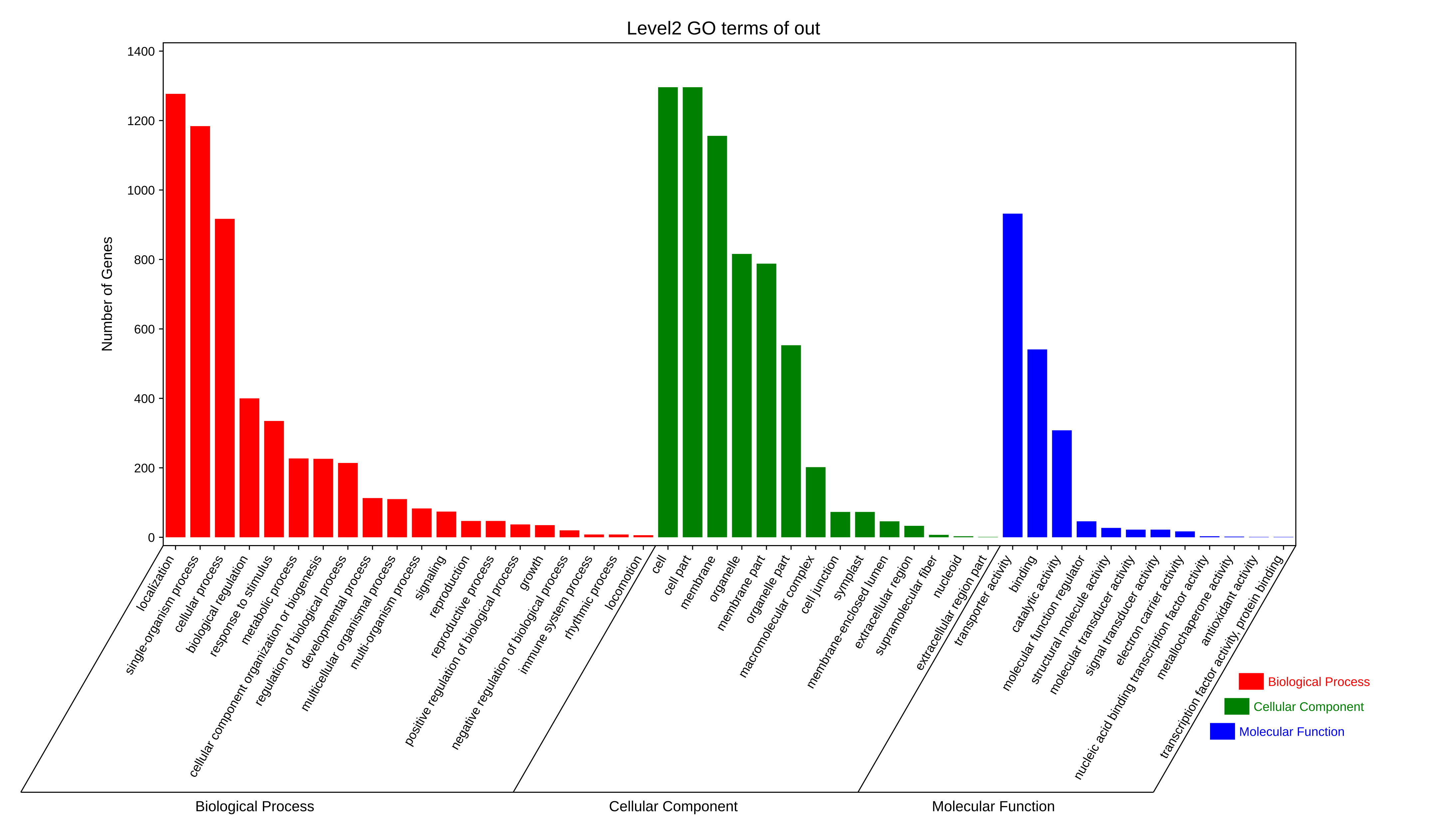


**Fig. S5 GO enrichment of 1,552 genes with "transport" function.**


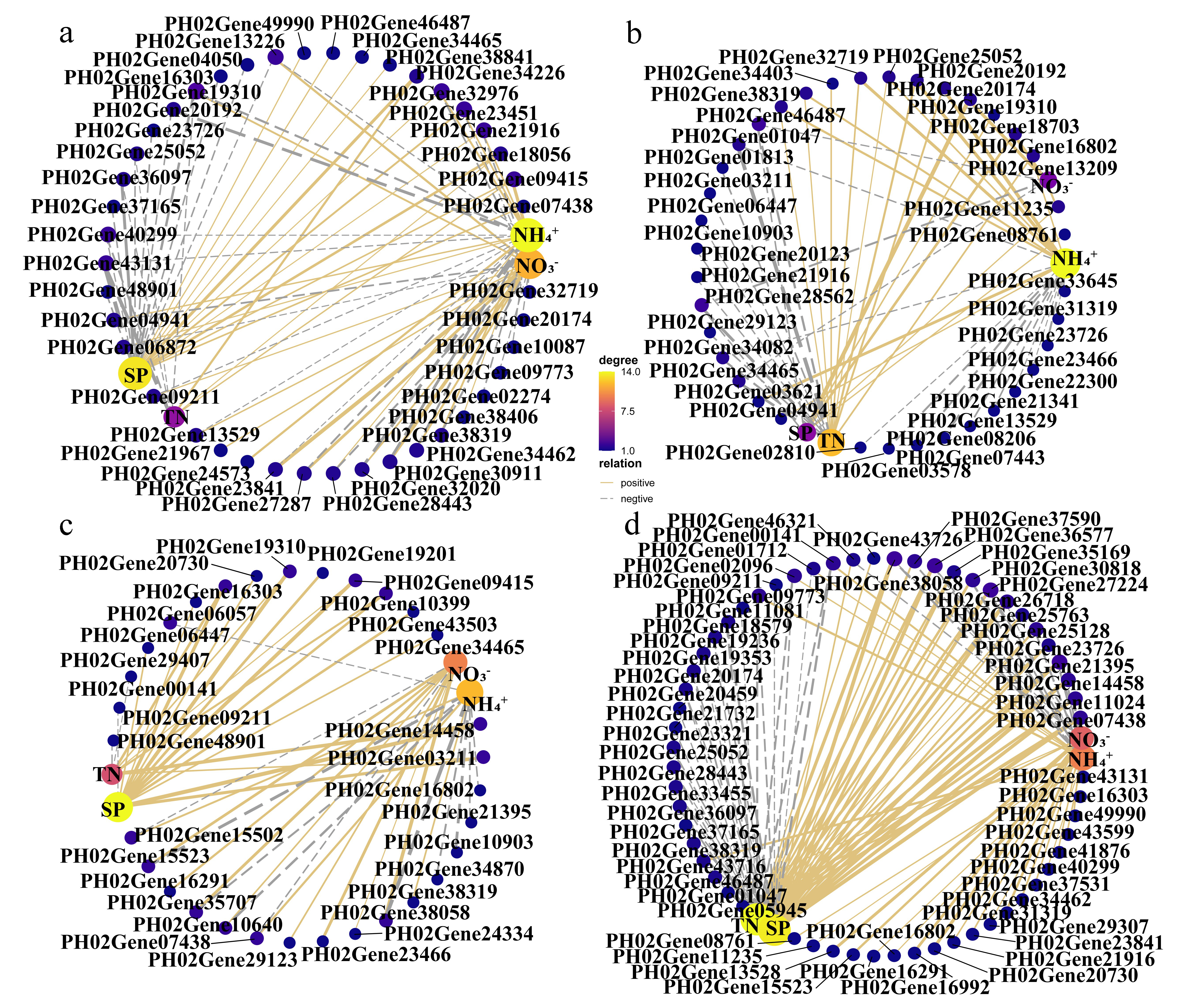


**Fig. S6 Correlation network between the expression of nitrogen transport genes and physiological traits of leaf (a), culm (b), rhizome (c) and shoot (d).**


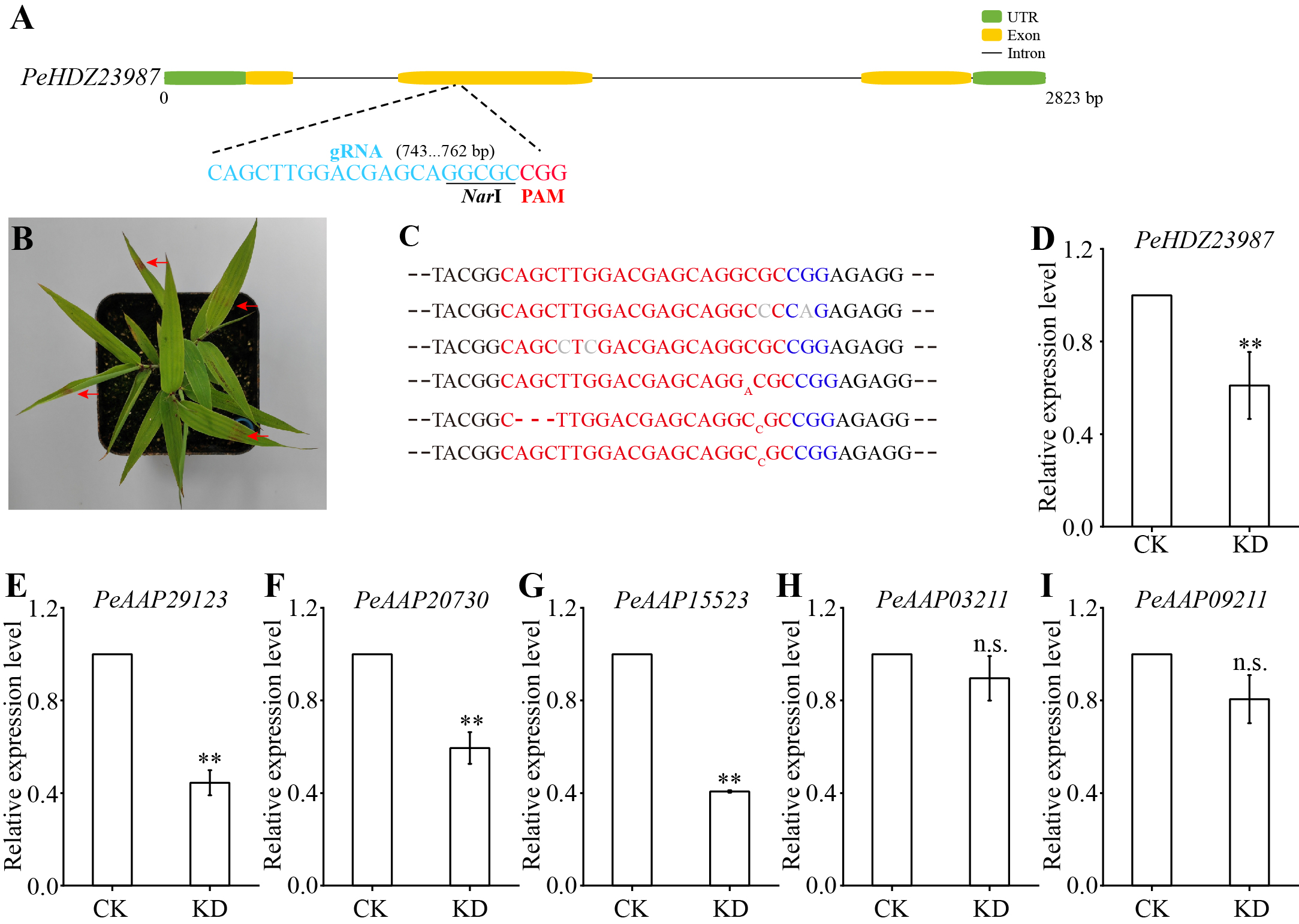


**Fig. S7 In-planta gene editing in Moso bamboo leaves**. (A) Schematic diagrams of sgRNA designs of *PeHDZ29387*. (B) In-planta gene editing in Moso bamboo seedlings for 7 days. (C) Deep sequencing results of the *PeHDZ29387* fragments. Portions of sequences in red, blue, and grey indicate the target sites, PAM, and insertions, respectively. The red dashes indicate deleted nucleotides. (D-I) The relative expression levels of *PeHDZ29387* and five *PeAAP*s. Significant differences are calculated by Student’s t-tests (**, *P* < 0.01; n.s., *P* > 0.05).
